# Exosomes derived from statin-modified bone marrow dendritic cells increase thymus-derived natural regulatory T cells in experimental autoimmune myasthenia gravis

**DOI:** 10.1186/s12974-019-1587-0

**Published:** 2019-11-03

**Authors:** Peng Zhang, Ru-Tao Liu, Tong Du, Chun-Lin Yang, Yu-Dong Liu, Meng-Ru Ge, Min Zhang, Xiao-Li Li, Heng Li, Ying-Chun Dou, Rui-Sheng Duan

**Affiliations:** 1 Department of Neurology, Shandong Provincial Qianfoshan Hospital, Shandong University, Jinan, 250014 People’s Republic of China; 2 Department of Neurology, the First Affiliated Hospital of Shandong First Medical University, Jinan, 250014 People’s Republic of China; 3 College of Basic Medical Sciences, Shandong University of Traditional Chinese Medicine, Jinan, 250355 People’s Republic of China

**Keywords:** Exosomes, Thymus, Experimental autoimmune myasthenia gravis (EAMG), Foxp3^+^ natural regulatory T cells, Aire

## Abstract

**Background:**

The thymus plays an essential role in the pathogenesis of myasthenia gravis (MG). In patients with MG, natural regulatory T cells (nTreg), a subpopulation of T cells that maintain tolerance to self-antigens, are severely impaired in the thymuses. In our previous study, upregulated nTreg cells were observed in the thymuses of rats in experimental autoimmune myasthenia gravis after treatment with exosomes derived from statin-modified dendritic cells (statin-Dex).

**Methods:**

We evaluated the effects of exosomes on surface co-stimulation markers and Aire expression of different kinds of thymic stromal cells, including cTEC, mTEC, and tDCs, in EAMG rats. The isolated exosomes were examined by western blot and DLS. Immunofluorescence was used to track the exosomes in the thymus. Flow cytometry and western blot were used to analyze the expression of co-stimulatory molecules and Aire in vivo and in vitro.

**Results:**

We confirmed the effects of statin-Dex in inducing Foxp3^+^ nTreg cells and found that both statin-Dex and DMSO-Dex could upregulate CD40 but only statin-Dex increased Aire expression in thymic stromal cells in vivo*.* Furthermore, we found that the role of statin-Dex and DMSO-Dex in the induction of Foxp3^+^ nTreg cells was dependent on epithelial cells in vitro.

**Conclusions:**

We demonstrated that statin-Dex increased expression of Aire in the thymus, which may further promote the Foxp3 expression in the thymus. These findings may provide a new strategy for the treatment of myasthenia gravis.

## Introduction

Myasthenia gravis (MG) is a chronic autoimmune disorder of neuromuscular transmission resulting in muscle weakness. The thymus plays a major role in the pathogenesis of MG with anti-acetylcholine receptor (AChR) antibodies. Sixty-five percent of patients with MG are found to have thymic dysplasia and another 10% of cases are associated with thymoma [1]. Naturally occurring thymus-derived regulatory T (nTreg) cells are generated in the thymus and are key players in the suppression of immune response. Several reports have illustrated the decreased amount and function of thymic nTreg cells in MG patients [2, 3]. This defect in nTreg cells attracts peripheral B cells and activated T cells, which maintain a chronically inflammatory thymic environment [4]. Such deleterious effects highlight the importance of nTreg cells function in the pathogenesis of MG.

Multiple factors can influence the development of thymic nTreg cells. Differentiation of nTreg cells is dependent on thymic stromal cells, which can be subdivided into thymic epithelial cells (TEC) and thymic dendritic cells (tDCs). There are two main populations of TEC based on different cell surface markers and their location in the thymus [5]. Medullary TEC (mTEC) are defined by their binding capacity to the lectin *Ulex europaeus* Agglutinin I (UEA-I), while cortical TEC (cTEC) are recognized by anti-aminopeptidase A (APA) expression. mTEC and cTEC possess distinct functions to maintain balanced microenvironments [6]. Developing thymocytes are positively selected by cTEC and subsequently negatively selected by mTEC or tDCs [7]. On the other hand, nTreg cells are selected at the double-positive (DP) stage through interaction with the MHC class II-expressing cells within the thymic medulla, where they begin to express Foxp3 and differentiate into Foxp3^+^ nTreg cells [8].

Molecular factors like the autoimmune regulator (Aire) and CD80/86 interactions can also influence thymic nTreg cells differentiation. An autoimmune regulator called Aire is a transcriptional regulator that is highly expressed in a subset of mTECs. The function of Aire is to maintain the thymic structure and enable thymic expression of tissue-restricted antigens (TRA). TRA are presented to thymocytes by mTEC and tDC and drive self-reactive thymocytes to differentiate into Treg cells, which is essential for the maintenance of self-tolerance. Mutations in *Aire* lead to multi-organ autoimmunity in both mice and humans [9]. In an *Aire* knockout (*Aire*^*−*^*/*^*−*^) murine model, the mice displayed changes in their thymic structure and exhibited a decrease in peripheral regulatory T cells [10, 11]. Moreover, the differentiation of Treg cells requires not only the recognition of antigen/major histocompatibility complexes by the T cell receptor but also cooperation between CD28-CD80/86 and CD40-CD40L co-stimulatory interactions [12, 13]. Together, this indicates that not only cellular mechanisms including TEC and DCs but also molecular factors including MHC II, CD80/86, CD40, and Aire, influence the generation of thymic nTreg cells.

Exosomes, which are extracellular vesicles ranging from 30 to 150 nm in diameter [14, 15], have been implicated in many aspects of autoimmune disease such as multiple sclerosis (MS) [16, 17], rheumatoid arthritis (RA) [18] and systemic lupus erythematosus (SLE) [19], as well as in immune regulation involved in functions of T cells, B cells, DCs and so on. Since exosomes are more stable and easier to store, they have attracted more attention in recent years. Previously, we have reported that statins had the ability to induce tolerogenic DCs that displayed the potential to regulate both cellular and humoral immunity to relieve clinical symptoms in experimental autoimmune myasthenia gravis (EAMG) [20, 21]. We also used ultra-centrifugation to obtain statin-DC-derived exosomes (statin-Dex) or DMSO-DC-derived exosomes (DMSO-Dex). We demonstrated the ability of statin-Dex to ameliorate the clinical symptoms of EAMG rats. These therapeutic effects were associated with upregulated levels of Foxp3^+^ nTreg cells in both the thymus and peripheral lymphoid organs [22]. Since nTreg cells played a vital role in immune-regulatory effects of statin-Dex, we wondered if the thymus was the predominant target in the process.

In this study, we explored how statin-Dex upregulates nTreg cells in the thymus in EAMG. We found that statin-Dex increased expression of Aire in the thymus in vivo. Furthermore, statin-Dex promoted the development of Foxp3^+^ nTreg cells among thymocytes with the existence of thymic stromal cells in vitro*.* Taken together, we demonstrated that statin-Dex increased expression of Aire in the thymus, which may further promote the Foxp3^+^ expression in the thymus.

## Materials and methods

### EAMG induction and exosome administration

Female Lewis rats, 6–8 weeks old, were purchased from Vital River Corporation (Beijing, China) and housed at the animal facility of the Institute. Food and water were provided ad libitum. All animal procedures were conducted in strict accordance with the institutional ethics committee. Animals were euthanized via deep anesthesia using isoflurane. EAMG models were induced by a subcutaneous immunization at the base of the tail (two sites) with 75 μg of AchR 97–116 peptide (from China Peptides Co., Ltd.; Shanghai, China), emulsified in Complete Freud Adjuvant containing 1 mg *Mycobacterium tuberculosis*, in a total volume of 200 μl.

Animals were divided into three groups. One group of rats was used as PBS control; the other two groups received exosomes (10 μg/rat) derived from either DMSO or statin-treated BMDCs. The exosomes were administered on days 5, 10, and 15 p.i. The rats were sacrificed on day 20 p.i.

### The culture of bone marrow dendritic cell and isolation of exosomes

To isolate and culture BMDCs, tibias and femurs were removed from 6- to 8-week-old Lewis rats. The whole bone marrow was flushed out and filtered through a 70-μm Cell Strainer. After lysis of erythrocytes, cells were re-suspended in RPMI 1640 medium containing 10% (v/v) fetal bovine serum, 1% (v/v) penicillin-streptomycin (Gibco, Invitrogen Ltd., Paisley, UK) in the presence of 10 ng/ml recombinant rat (rr) GM-CSF (Peprotech, Rocky Hill, NJ, USA), and 10 ng/ml rrIL-4 (Peprotech). After a 72-h incubation, non-adherent cells were gently removed and adherent cells were further cultured. On day 7, non-adherent and loosely adherent cells were harvested and incubated with atorvastatin dissolved in dimethylsulfoxide (DMSO; Sigma-Aldrich, St. Louis, MO, USA, final concentration 10 μM) or DMSO (only in medium) with 10% FBS depleted of contaminating vesicles and protein aggregates for another 48 h at 37 °C and 5% CO_2_, before they were centrifuged at 300×*g* for 5 min. The supernatant was collected for exosomes isolation.

Exosomes were isolated and characterized as previously described. Briefly, the supernatant was centrifuged at 2000×g for 10 min and 10,000×*g* for 30 min to remove whole debris and large vesicles. The resultant supernatant fluid was then centrifuged at 100,000×*g* for 70 min (Beckman Coulter. Inc., IN, USA). Exosome pellets were rinsed with PBS and re-centrifuged at 100,000×*g* for 70 min. Finally, exosomes were suspended in sterile PBS and quantified by the K5600 MicroSpectroPhotoMeter (Beijing Kaiao Technology Development Co. Ltd., Beijing, China). Exosomes were stored at − 20 °C.

### Identification of exosomes

For transmission electron microscopy analysis, carbon-coated copper grids were placed in the exosome suspensions fixed with 2% paraformaldehyde overnight. The grids were then negatively stained by 2% phosphotungstic acid for 5 min and air-dried for 1 min at room temperature. The exosome samples were visualized by a Tecnai 20 U-TWIN operated at 80 kV (Philips, Nederland).

The exosome size was measured by dynamic light scattering (DLS). Briefly, exosomes (1 μg) were re-suspended in 1 ml-filtered PBS at pH 7.4. The sizes of the particles were analyzed by DLS Nano sizer (Nano-ZS; Malvern, UK).

### Tracking analysis in the thymus

Purified exosomes were labeled with membrane-targeted red fluorescent dye PKH26 (Sigma-Aldrich). In brief, 20 μl of exosomes suspended in 100 μl of Diluent C mixed with an equal volume of Diluent C containing 0.4 μl PKH26 dye and was incubated at 4 °C for 5 min. The labeling reaction was stopped by the addition of 10% FBS in PBS. The labeled exosomes were purified by centrifugation at 4000×g for 30 min, followed by ultrafiltration (0.22 μm filter; Millipore, Billicera, MA, USA) three times and were finally re-suspended in PBS. For uptake studies, 10 μg of labeled exosomes suspended in 300 μl of PBS were injected to EAMG rats. Following 3 days of intravenous injection, thymuses removed from rats in different groups were visualized with a fluorescence microscope to determine the distribution of exosomes.

### Preparation of thymic stromal cells

The thymic stromal cells were isolated as described previously [23]. In brief, thymuses were dissected from freshly killed rats and trimmed of fat and connective tissue. Tissues were gently agitated on a 70-μm filter with a vitreous stirrer. Thymocytes were collected under the filter. The resulting thymic fragments were transferred into a petri dish and then incubated in 1 mg/ml collagenase D with 0.1% (w/v) DNAse I (both from Roche Applied Science, Indianapolis, IN, USA) at 37 °C for 10 min. Enzyme mixtures with isolated cells were removed and replaced with the fresh mixture for further incubation. After another 10 min of digestion, isolated cells were removed and collagenases/dispase were added. During the digestion, gentle mechanical agitation was performed with a 26G needle to break up any remaining aggregates. Cells were pooled and resuspended in 5 mM EDTA buffer and incubated for 10 min at 4 °C to disrupt rosettes, followed by centrifugation at 300×*g* for 8 min. Cells were then passed through a 70-μm filter to remove clumps. Both thymocytes and thymic stromal cells were counted and prepared for antibody labeling.

### In vitro Treg cells induction experiments

To determine the functions of exosomes on the differentiation of thymic Treg cells, thymocytes were cultured with exosomes and TECs, exosomes and BMDCs, or exosomes only. After digestion described above, thymic CD45^−^ stromal cells were labeled and sorted by FACS. Thymocytes (2 × 10^6^/ml) and BMDCs (2 × 10^5^/ml) or CD45^−^ stroma cells (2 × 10^5^/ml) were suspended in the medium with 20 μg/ml exosomes. This was added into 96-well plates under stimulation with 5 μg/ml of IL-2 (PeproTech, NJ, USA), 1 μg/ml anti-CD3 (PeproTech), and 1 μg/ml anti-CD28 (PeproTech) mAb. After 2 days, the cells were collected and the percentages of CD4^+^Foxp3^+^ T cells were determined by FACS.

### FACS

For cell surface marker analysis, thymic TEC and DCs were stained using conventional methods. Antibodies were all mouse monoclonal antibodies unless specified. CD86 (24H), OX62 (OX-62), and CD45 (OX-1) were obtained from Biolegend (San Diego, CA, USA). CD4 (OX35), MHC II (HIS19), CD40 (HM40-3), and CD80 (3H5) were purchased from eBioscience (San Diego, CA, USA). Unconjugated APA (goat polyclonal IgG) was obtained from Santa Cruz (United States) and the secondary antibody FITC or Alexa Fluor 647 conjugated donkey anti-goat IgG was purchased from Abcam (Cambridge, MA, USA). UEA-1 was purchased from Vector Laboratories (Burlingame, CA, USA).

For nTreg cells, thymocytes were stained with specific Abs against rat CD4, CD8 (eBioscience) and CD25 (OX39, Invitrogen) before being fixed and permeabilized with Perm/Fix solution (eBioscience). Finally, an antibody specific for Foxp3 (rat monoclonal antibody, FJK-16S, eBioscience) was added for 30 min in the dark.

### Immunofluorescence

Thymus tissues were cryo-sectioned and processed for the immunofluorescence (IF) assay. Sections (8 μm) were fixed in cold acetone and incubated in 0.5% bovine serum albumin (BSA) (Solarbio, Beijing, China). Samples were incubated with primary antibody Aire (Santa Cruz) at 4 °C overnight. Afterward, sections were incubated with secondary fluorescently tagged antibody protected from light. Images were acquired on an Olympus FSX100 (Tokyo, Japan) at different magnifications. The fluorescence microscopy examination was performed in a blinded manner.

### Western blotting

A western blot was performed to determine thymic Aire from thymus tissues. Proteins were separated on a gel and transferred onto a PVDF membrane. Anti-Aire (Santa Cruz) and anti-GADPH (Zhongshan Goldenbridge Biotechnology, Beijing, China) were used as primary antibodies; horseradish peroxidase-coupled goat anti-mouse or goat anti-rabbit (Zhongshan Goldenbridge Biotechnology) as secondary antibodies. The immunoreactive proteins were visualized using a FluorChem E Imager (Protein-Simple, Santa Clara, CA, USA). Bands were quantified with ImageJ software (National Institutes of Health, Bethesda, MD, USA).

### Statistical analysis

Statistical tests were performed using GraphPad Prism 7.0 (GraphPad Software, San Diego, CA, USA). Differences between controls and experimental groups were determined with one-way ANOVA and followed by the Bonferroni test as a post hoc test. *P* < 0.05 was considered statistically significant.

## Results

### Identification of exosomes derived from DCs culture medium

**Fig. 1:**
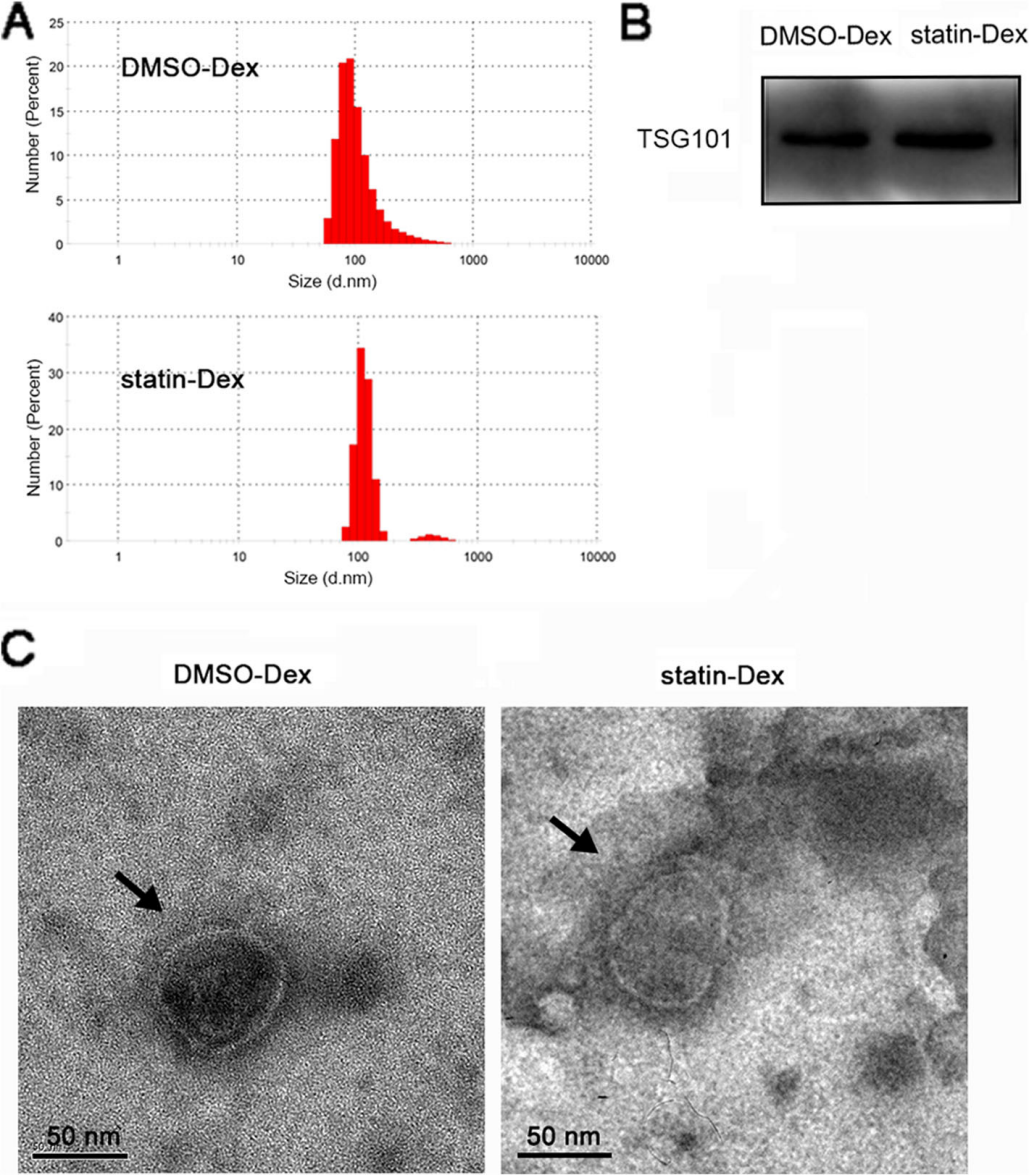
Characterization of DMSO-Dex and statin-Dex. **a** DLS Nano sizer analysis showed size distribution of exosomes from the DMSO-Dex and the statin-Dex groups, ranging from 70 to 140 nm (mainly from 80 to 120 nm). **b** TSG101 expression with size 49 kDa in DMSO-Dex and statin-Dex. **c** Representative images of the exosome from the DMSO-Dex and the statin-Dex groups under transmission electron microscopy (scale bar 50 nm)

For isolation of exosomes, extracellular vesicles from BMDC culture supernatants were obtained by ultracentrifugation (approximately 3–8 μg particles from 1 ml fluid). According to the proof of morphology, the particles were identified by DLS instruments (Fig. 1a), western blotting (Fig. 1b), and electron microscopy (Fig. 1c), respectively. The diameters of particles in the DMSO-Dex and the statin-Dex groups were primarily distributed in the range of 70–140 nm. The morphology observed under an electron microscope was shown to be intact, with a diameter of about 100 nm. To confirm the exosome-enriched fractions, TSG101 (a well-established marker for exosomes) was determined by western blot. Exosomes from both the DMSO-Dex and the statin-Dex groups expressed the TSG101 with a size of 49 kDa.

### Exosomes are distributed mostly in the cortex of the thymus

**Fig. 2:**
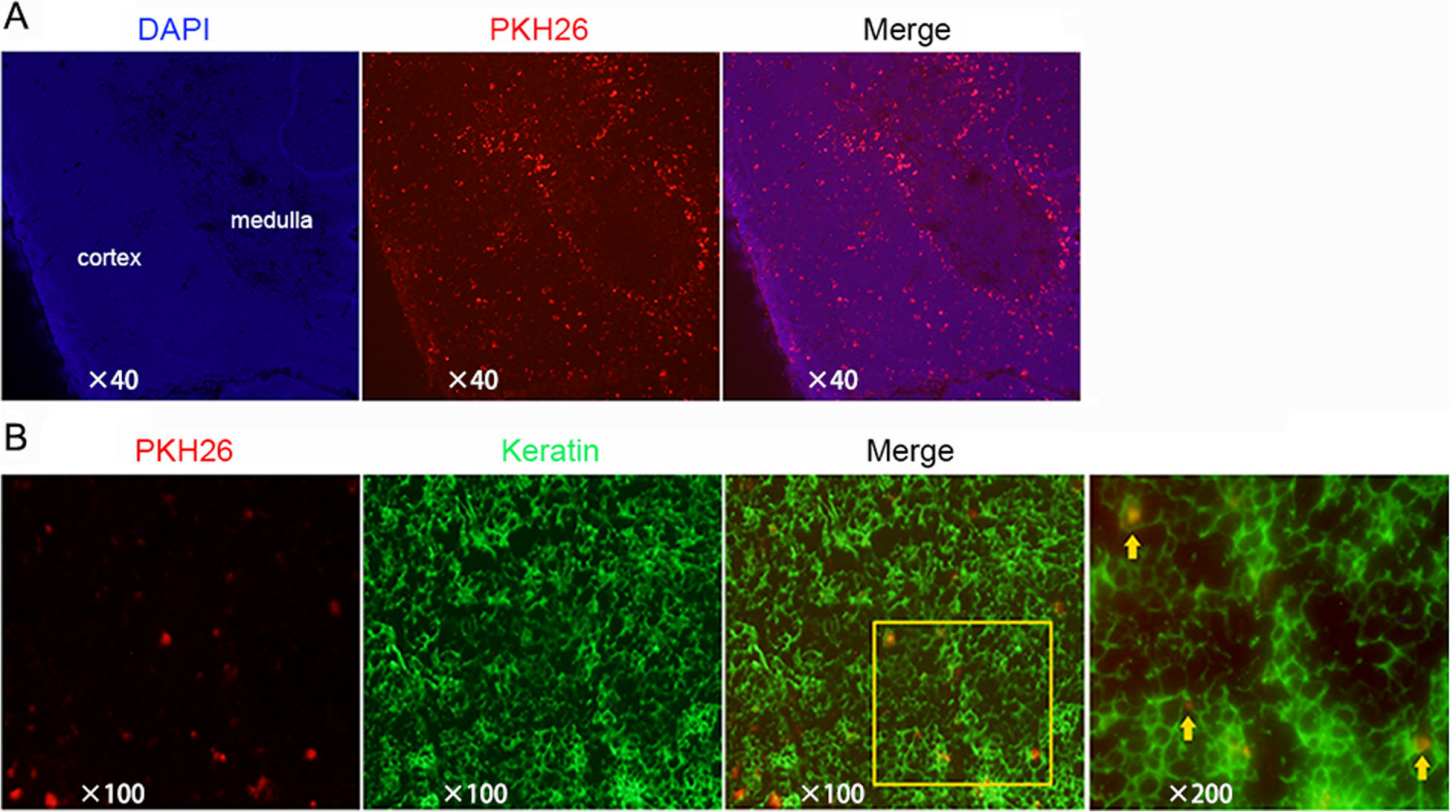
Delivery of exosomes labeled with PKH26 in the thymus. **a** Cryostat sections of the thymus from rats receiving PKH26-labeled statin-Dex (red) were stained with DAPI (blue). **b** Cryostat section of the thymus stained with stromal cell-specific keratin (green) from rats receiving an intravenous injection of PKH26-labeled statin-Dex (red)

In our previous study, exosomes were found in the thymus, lymph nodes and spleen except the liver on day 3 after injection [22]. To further verify that exosomes entered and functioned in the thymus, exosomes were labeled with PKH26 in vitro and then injected into EAMG rats via the tail vein. Three days after the injection, the rat thymuses were removed and sectioned to observe the distribution of exosomes under a microscope. As shown in Fig. 2a, exosomes were mainly located at the cortex within the thymus. There were more PKH26-labeled exosomes around medulla because they were spilled from the blood vessels, which are rich in the cortico-medullary junction [24]. There is no difference in the distribution between the DMSO-Dex group and the statin-Dex group. Furthermore, exosomes were shown to be engulfed by the keratin positive thymic epithelial cells in the cortex (Fig. 2b).

### Statin-Dex increases Foxp3 expression in thymocytes

**Fig. 3:**
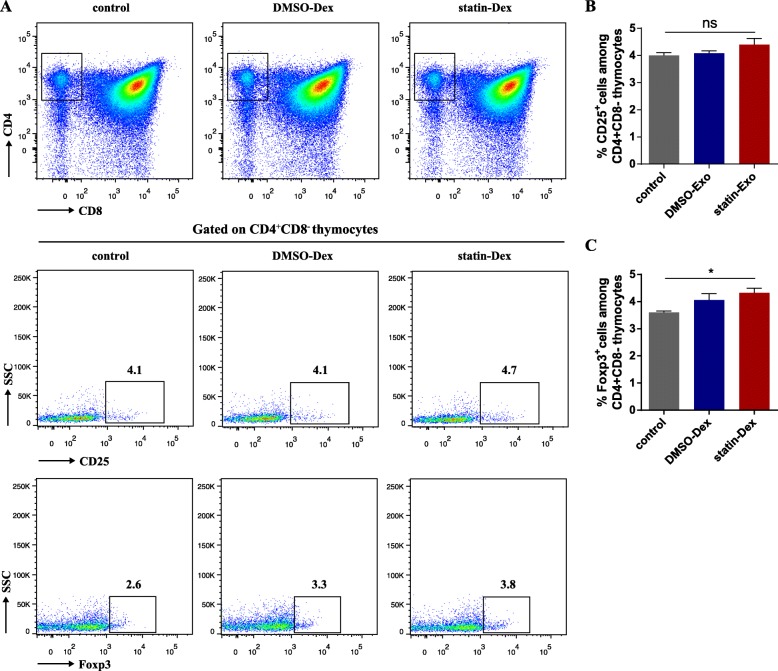
Statin-Dex promoted thymic nFoxp3^+^ Treg cell differentiation. **a** The representative gating strategy of CD4, CD8, and representative dot plots of CD25 expression, and Foxp3 expression are shown. Percentages of CD25 (**b**) and Foxp3 expression (**c**) among CD4^+^CD8^−^ thymocytes were analyzed. Data were presented as mean ± SEM from two independent experiments (*n* = 5 in control group; *n* = 6 in DMSO-Dex and statin-Dex groups, *p < 0.05). The significance of the differences was evaluated by ANOVA, followed by Bonferroni test as a post hoc test

Our previous study reported that statin-Dex induced an increase of Foxp3 expression in the thymus and peripheral Foxp3^+^ nTreg cells in lymph nodes. In this study, we focused on its effects on thymic nTreg cells. In the early stage of EAMG disease, on day 20 p.i., the percentage of nTreg cells in the thymus was detected by FACS. As shown in Fig. 3b, there were no statistical differences in CD4^+^CD8^−^CD25^+^ thymocytes (thymic nTreg cells) among the three groups. However, the percentage of Foxp3^+^ nTreg cells among CD4^+^CD8^−^ thymocytes was significantly higher in the statin-Dex group than in the PBS-treated control group (Fig. 3c).

**Fig. 4:**
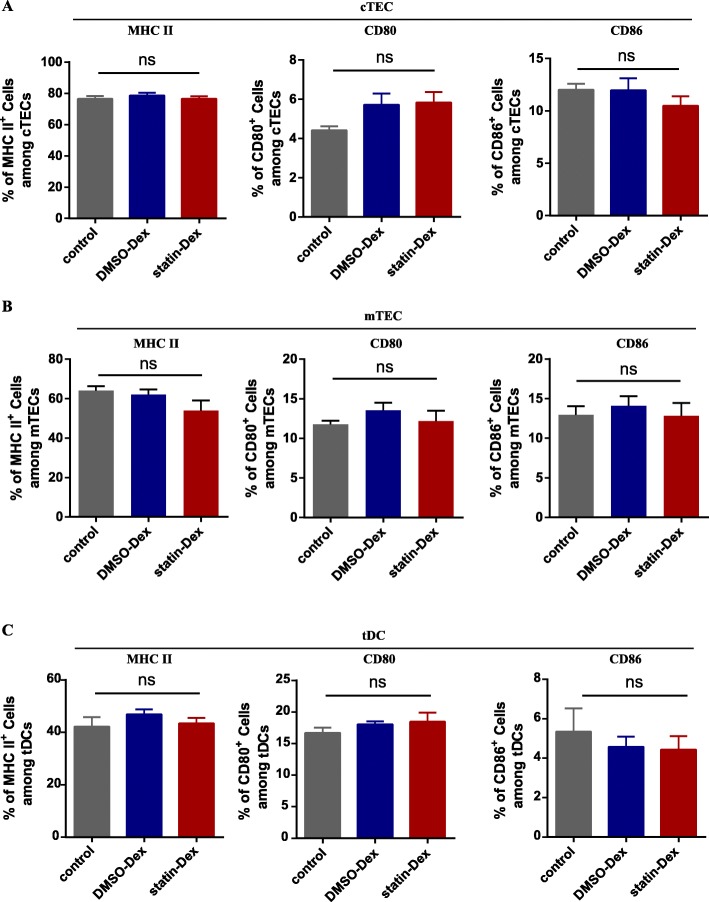
Expression of MHC II, CD80, and CD86 on cTEC (CD45^−^APA^+^), mTEC (CD45^−^UEA-1^+^), and tDCs (OX62^+^). After digestion, single thymic stromal cells were obtained and the expression of MHC II, CD80, and CD86 on cTEC (**a**), mTEC (**b**), and tDCs (**c**) were examined by flow cytometry, respectively (ns means no significance). Data were representative of two independent experiments (*n* = 5 in the control group; *n* = 6 in DMSO-Dex and statin-Dex groups)

### MHC II and B7 molecules remain unchanged in the control group, the DMSO-Dex group, and statin-Dex groups

On day 20 p.i., thymuses were removed from rats in the control group, the DMSO-Dex group, and the statin-group. After thymocytes removal, TEC and tDCs were obtained as described. tDCs were defined as OX62 positive cells, cTEC as CD45^−^APA^**+**^**,** and mTEC as CD45^−^ UEA1^**+**^. To investigate whether co-stimulatory molecules of thymic stromal cells were affected by exosomes, the expression levels of MHC II, CD80, and CD86 were examined by flow cytometry. However, there were no significant differences among the three experimental groups (Fig. 4 a-c), suggesting that MHC II and B7 molecules may not be involved in the regulation of Foxp3^+^ nTreg cells by exosomes.

### DC-derived exosomes upregulate CD40 expression on thymic medullary epithelial cell and thymic DCs

**Fig. 5:**
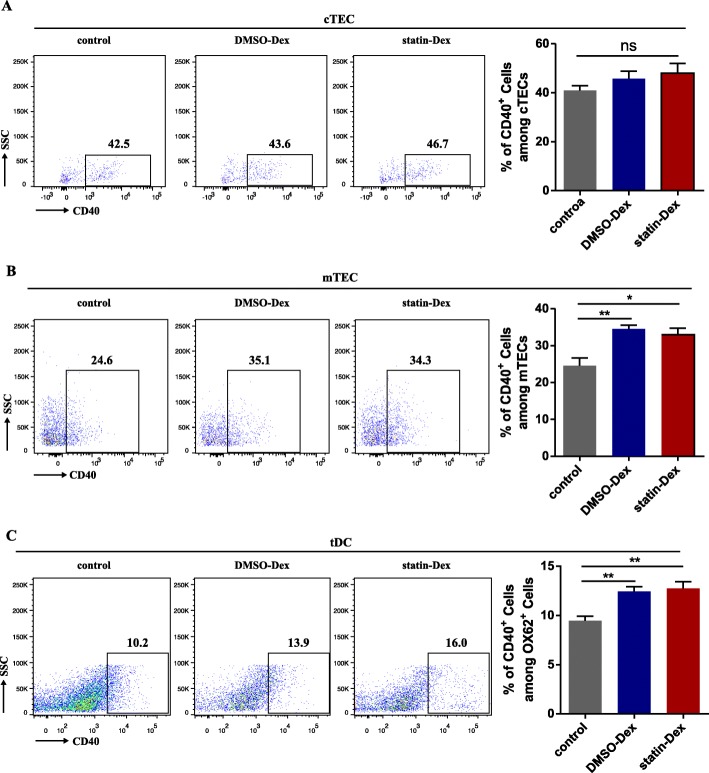
Exosomes increased the expression of CD40 in the thymus. There is no change in the expression of CD40 on cTEC among the three experimental groups (**a**). Both DMSO and statin-treated DC-derived exosomes had significantly increased CD40 expressions on mTEC (**b**) and tDCs (**c**) compared with the PBS treated control group (*p < 0.05 and **p < 0.01). Data were presented as mean ± SEM from two independent experiments (*n* = 5 in the control group; *n* = 6 in DMSO-Dex and statin-Dex groups). The significance of the differences was evaluated by ANOVA, followed by Bonferroni test as a post hoc test

CD40 expression in the thymus is also essential for promoting the development of thymic Foxp3^+^ nTreg cells. As a result, the CD40 level of the thymic stromal cells was further examined. Compared to the control group, both DMSO-Dex and statin-Dex increased the expression of CD40 on the mTEC and tDCs, but there was no alteration of CD40 expression on the cTEC in the thymus (Fig. 5a–c).

### Statin-Dex increase Aire in the thymic medulla

**Fig. 6:**
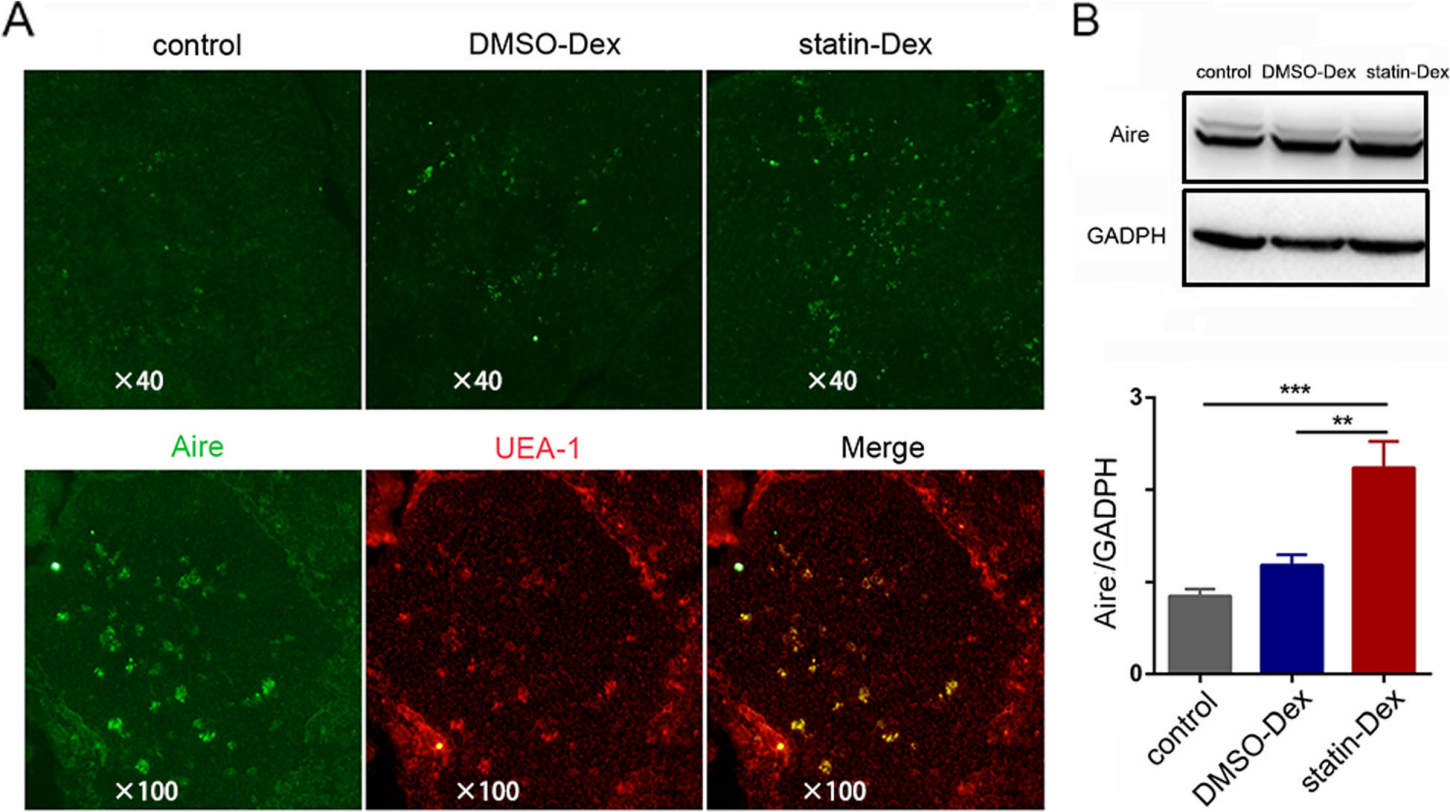
Statin-Dex upregulates the expression of Aire in the thymus. **a** Frozen tissue sections of thymus were stained with antibodies to Aire (green) and UEA-1 (red) to examine the expression of Aire in mTEC. **b** Aire expression analysis in the thymus by western blot (**p < 0.01 and ***p < 0.001). Data were presented as mean ± SEM (*n* = 5 in the control group; *n* = 6 in DMSO-Dex and statin-Dex groups). The significance of the differences was evaluated by ANOVA, followed by Bonferroni test as a post hoc test

It has been reported that CD40 contributes to the expression of Aire in thymic medullary epithelial cells and Aire is critical to the nTreg cell differentiation in the thymus [25, 26]. On day 20 p.i., the thymuses were obtained and Aire expression was examined by immunofluorescence. Immunofluorescence results demonstrated an obviously increased Aire expression in thymic medullary epithelial cells in the statin-Dex group when compared to both the control group and the DMSO-Dex group (Fig. 6a). Increased Aire expression in the thymus in the statin-Dex group was further confirmed by the western quantitative analysis compared to the control group and the DMSO-Dex group (Fig. 6b).

### DC-derived exosomes promote the development of Foxp3^+^ Treg cells among thymocytes by TEC in vitro

**Fig. 7:**
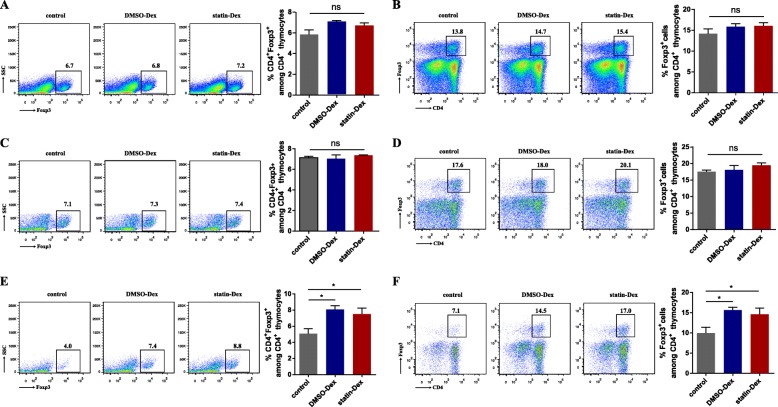
CD45^−^ stromal cells promote the thymic Foxp3^+^ Treg cells differentiation in vitro. Frequencies of CD4^+^Foxp3^+^ Treg cells among total thymocytes and Foxp3^+^ Treg cells among CD4^+^ thymocytes after 2 days of incubation with PBS control, DMSO-Dex, and statin-Dex, respectively (**a**, **b**). Thymocytes were cultured with BMDCs (**c**, **d**) or CD45^−^ stromal cells (**e**, **f**) (at a ratio of 1:10) with PBS control, DMSO-Dex and statin-Dex, respectively. (*p < 0.05). Data were representative of three independent experiments. The significance of differences was evaluated by ANOVA, followed by Bonferroni test as a post hoc test

Thymocytes were cultured with DMSO-Dex, statin-Dex, and PBS respectively and the percentages of Foxp3^+^ thymocytes were measured 48 h later. There was no difference in the percentages of Foxp3^+^ Treg cells among the three experimental groups (Fig. 7a, b), which indicates that exosomes may not directly affect Foxp3^+^ Treg cells differentiation in the thymus. To assess whether the impact of exosomes on the thymic Treg cell differentiation was dependent on DCs, BMDCs, and thymocytes (at a ratio of 1:10) were co-cultured with DMSO-Dex, statin-Dex, and PBS, respectively. After 48 h of cultivation, no differences in the percentages of Foxp3^+^ Treg cells in the three experimental groups were observed (Fig. 7c, d), which suggested that exosomes could not promote Foxp3^+^ Treg cell expansion through regulation of DCs. CD45^−^ cells (mainly TEC) were isolated and co-cultured with thymocytes (at a ratio of 1:10) in the presence of PBS, DMSO-Dex, and statin-Dex. The percentages of both CD4^+^Foxp3^+^ Treg cells among total thymocytes (Fig. 7e) and Foxp3^+^ Treg cells among CD4^+^ thymocytes (Fig. 7f) were higher in the two exosome groups compared to the control. Taken together, these data provided strong evidence that upregulated Foxp3^+^ Treg cells by exosomes in thymocytes is mainly mediated by TEC.

## Discussion

Exosomes are nano-bioactive vesicles that can be secreted by almost all types of cells, and may contain biomarkers from the source cells. Exosomes derived from DCs carry MHC II and T cell co-stimulatory molecules on their surface, suggesting that they could play regulatory roles in immune response. It has been demonstrated that DC-derived exosomes could be either immune-stimulatory or suppressive depending on the type and stage of maturation of the DCs [27–29]. Immature DC-derived exosomes display a tolerance activity in a mouse model of EAMG [30]. Exosomes derived from microRNA-146a overexpressing DCs suppress ongoing EAMG in a partly dose-dependent manner [31]. In our study, to some extent, both DMSO-treated and statin-treated DCs showed immature characteristics due to lack of stimulation by LPS or CpG. The effect of DMSO-Dex is due to the fact that there were some immature DCs in the culture system of DC treated with DMSO*.* This may account for the similar effect of DMSO-Dex and statin-Dex on the CD40 expression of mTEC and tDCs in vivo and the differentiation of Foxp3^+^ Treg cells in vitro. Our previous data showed that exosomes derived from statin modified DCs displayed a lower level of MHC II than exosomes from DMSO modified DCs, indicating a more immature phenotype of statin-modified DCs [22]. However, only statin-Dex upregulate the expression of Aire in the thymus, which accounts for its effect on the differentiation of nTreg in vivo. In our previous study, statin-treated DCs from EAMG rats inhibited the antigen-specific lymphocyte proliferation in the presence of peptide AchR 97-116, whereas exosomes derived from these statin-DCs did not [21, 22]. Therefore, the exosomes from the DC of healthy rats in the present study were not antigen-specific. In the present study, exosomes derived from statin-DC increased thymic Aire expression and induced the differentiation of thymic Foxp3^+^ nTreg cells in EAMG, which played an essential role in central immune tolerance. It is reasonable to hypothesize that statin-Dex would also increase thymic Aire expression in healthy animals but it is more meaningful in the autoimmune diseases.

The thymus is a primary lymphoid organ required to induce and maintain central tolerance by clonal deletion of self-reactive thymocytes or by driving nTreg differentiation. nTreg cells generated in the thymus can migrate into the peripheral and suppress autoimmune responses. They play an important role in the maintenance of immune tolerance and homeostasis. Several scientific research have tried to explore the process in which nTreg cells reach functional maturity within the thymus. The generation of nTreg cells is under strict regulation and control in the thymus. Both mTEC and tDC are shown to be important for nTreg cells differentiation. And mTEC were considered to serve as the key accessory cell for nTreg selection. Our ex vivo data showed that exosomes can enhance Treg cell generation only in the presence of TEC, suggesting that TEC were essential for exosomes to upregulate the Foxp3 Treg cells.

Studies have shown that most Foxp3^+^ cells are mainly located in the medulla of thymus, which suggests the important role of mTEC in the development of nTreg cells [32, 33]. Mice with disrupt thymic medulla have decreased Foxp3 expression [34]. Furthermore, many studies emphasized the roles of Aire in regulating mTEC differentiation and Foxp3^+^ nTreg cells differentiation in the thymus [35–37]. Aire is predominantly expressed in mTEC and a negative selection of tissue-specific T cells during intrathymic development is aided by Aire. Depletion of Aire-expressing mTEC and tDCs are in accordance with a dramatic decrease of Foxp3^+^ nTregs in the thymus of Omenn syndrome patients. This suggests a role of Aire-expressing mTEC and/or thymic DCs in the differentiation of Foxp3^+^ nTreg cells [38]. Previous data have indicated a correlation between the patients with autoimmune polyendocrinopathy candidiasis ectodermal dystrophy (APECED) and Treg cells. This is characterized by the loss-of-function mutations in the *Aire* gene, and an impaired circulating Foxp3^+^ Treg population [39, 40]. The defect of Treg is not only the frequency of Tregs but also their levels of Foxp3 expression and function. The report has shown that abrogating thymic expression of Aire, via deletion of conserved non-coding sequence (CNS1) (a putative enhancer upstream of the *Aire* gene), leads to impaired terminal differentiation of the mTEC population and a reduction in the production of thymic nTreg cells [41]. Overall, the available evidence supports the essential effect of Aire in Foxp3^+^ nTreg cells development. Our data demonstrated that statin-Dex increased Aire expression in the thymic medulla, which may lead to the increase of Foxp3^+^ nTreg cells.

MHC II on the epithelial cells plays an essential role in the differentiation of nTreg in the thymus. The development of Treg cells is initially dependent on TCR-MHC II and CD28-CD80/CD86 interaction, which leads to the generation of thymic Treg cell precursors (pre-Treg cells). However, there were no differences among the three groups in the expression of MHC II as well as CD80 and CD86 molecules in our study. CD40 has been observed to be expressed on tDCs, cTEC, and mTEC [42]. It is a co-stimulatory molecule associated with the mature state of mouse tDCs and has been shown to affect levels of MHC II, CD80, and CD86 on APCs through interactions with CD40L [43]. CD40 has a role in the expansion of nTreg populations. Ablation of CD40 expression in tDCs results in a significant reduction in the number of nTreg cells. CD4^+^ thymocytes co-culture with CD40-deficient DCs fail to differentiate them into nTreg cells to a sufficient number in vitro [44]. Furthermore, it was reported that disruption of CD40 signaling would lead to a severe defect in mTEC. Importantly, expression of CD40 on either tDCs or TEC is sufficient to promote the development of Fxop3^+^ Treg cells [45]. Our study demonstrated that both exosome treatments upregulated CD40 expression in mTEC and tDCs but without any change for the expression of MHC II and B7 molecules in thymic stromal cells. Furthermore, only statin-Dex increased thymic Aire expression and thereafter thymic nTreg differentiation in vivo*.*

## Conclusion

We evaluated the effects of exosomes on surface co-stimulation markers and Aire expression of different kinds of thymic stromal cells, including cTEC, mTEC, and tDCs. It was established that the increased thymic Foxp3^+^ nTreg cells in statin-Dex treatment was correlated with upregulated Aire in mTEC in vivo. Statin-Dex promoted the development of Foxp3^+^ Treg cells among thymocytes with the existence of thymic stromal cells in vitro. All these data demonstrated that statin-Dex may induce central tolerance in EAMG, which may provide a new strategy in the treatment of myasthenia gravis.

## Data Availability

All data generated or analyzed during this study are available on request.

## Abbreviations

Aire: Autoimmune regulator
APA: Anti-aminopeptidase A
BMDC: Bone marrow dendritic cells
cTEC: Cortical thymic epithelial cells
DC: Dendritic cell
DMSO-Dex: Exosomes derived from DMSO-modified dendritic cells
EAMG: Experimental autoimmune myasthenia gravis
MG: Myasthenia gravis
MHC II: MHC class II
MS: Multiple sclerosis
mTEC: Medullary thymic epithelial cells
RA: Rheumatoid arthritis
SLE: Systemic lupus erythematosus
statin-Dex: Exosomes derived from statin-modified dendritic cells
tDC: Thymic dendritic cells
TEC: Thymic epithelial cells
TRA: Tissue-restricted antigens
Treg: Regulatory T cells
UEA-I: *Ulex europaeus* Agglutinin I
